# Determinants of Intergenerational Downward Mobility in the Netherlands

**DOI:** 10.1007/s11205-015-1066-7

**Published:** 2015-08-29

**Authors:** Lex Thijssen, Maarten H. J. Wolbers

**Affiliations:** Department of Sociology, Radboud University, P.O. Box 9104, 6500 HE Nijmegen, The Netherlands

**Keywords:** Downward mobility, Social stratification, The Netherlands, Occupational status

## Abstract

Several studies have shown that Dutch society has become more open in the last few decades as a result of increasing opportunities for upward social mobility. However, recently it has been observed that the likelihood of downward social mobility has increased for the youngest (male) birth cohorts in the Netherlands. Despite this recent finding, social stratification research has paid little attention to test explanations of downward mobility. This article tries to fill in this knowledge gap by testing three theoretical perspectives that aim to explain intergenerational downward occupational mobility of individuals. In addition, we examine historical developments to study whether the role of these explanations have changed over time. To test the predictive validity of these perspectives, we use data from the Family Survey Dutch Population 2009 (N = 1423). The empirical results, first of all, indicate that individuals who were born in younger birth cohorts are more likely to experience downward occupational mobility than individuals, who were born in older cohorts. We thus replicate earlier findings for the Netherlands. Secondly, we find that cognitive skills and, especially, educational attainment provide individuals significant protection against downward mobility. These findings are mainly in line with the meritocratic perspective. Thirdly, the results reveal that the role of the presumed explanations of downward mobility has not changed over time.

## Introduction

At the beginning of the twentieth century, most Western countries were experiencing a transition from agrarian to (post)industrialized society (Breen 2004). As a result, labour markets modernized, people experienced more prosperity, and meritocratic values became more widespread. Central theme in, for example, social democratic ideology is the idea that class systems in modern society are open and occupational positions are filled in on the basis of merit, like educational attainment, instead of social origin. To attain this ideal, governments improved access to (higher) education (Waslander and Bosman 1997). The increased enrolment in education, in combination with flourishing economies and a great(er) demand for high skilled labour have given more opportunities for people to experience upward social mobility.

Echoing these societal trends, social stratification sociologists became interested in research on the openness of societies (Blau and Duncan 1967; Breen 2004; Erikson and Goldthorpe 1992; Lipset and Bendix 1959). In the Netherlands, for example, research revealed that social origin is nowadays to a lesser extent associated with an individual’s educational attainment and occupational achievement than it used to be (de Graaf and Luijkx 1995; Ganzeboom and Luijkx 2004). In addition, although findings are inconsistent regarding whether education has become more important in predicting persons’ occupational position over time (de Graaf and Luijkx 1995; Ganzeboom and Luijkx 2004; Tolsma and Wolbers 2014), education is generally found to be the most important predictor of an individual’s occupational position nowadays (de Graaf and Ultee 1998). Hence, these findings suggest that a process from ascription to achievement has taken place in Dutch society.

The question is whether there are limits to this ongoing trend to societal ‘openness’ and upward mobility in the Netherlands? As a result of the increased educational participation of individuals, the educational level of the Dutch (working) population has risen considerably. However, because the upgrading of the occupational structure did not keep pace with the enormous educational expansion that took place, the demand–supply ratio for high skilled work has decreased, resulting into a process of ‘credential inflation’ in the Netherlands (Wolbers et al. 2001). As a consequence, high skilled workers are forced to accept jobs at a lower level than they actually obtained in education to avoid (long-term) unemployment. High educated workers displace middle educated workers, and eventually, middle educated workers push out low educated workers of employment. In other words, a process of ‘crowding out’ has taken place in recent years (Wolbers et al. 2001; Gesthuizen and Wolbers 2010). When this process is lasting, this could lead to a development in which an increasing number of individuals, despite their higher level of education attained, take an occupational position which is lower than that of their parents who attained their position in more advantageous labour market conditions. In other words, (young) people are more likely to become intergenerational downward occupational mobile nowadays.

It is important to make a distinction here between absolute and relative social mobility. Absolute mobility refers to the question how many individuals are in higher or lower social positions than their parents. Relative mobility rates describe the association in social class position between parents and their children. To determine this association adequately, changes in the occupational structure (between the two generations) need to be taken into account. Structural changes can have a strong impact on the total amount of mobility in society. Previous research showed that large part of the total mobility rate in the Netherlands can be ascribed to the increased demand for high skilled labour (Tolsma and Wolbers 2010). Both forms of social mobility occur at the same time. In this article, we focus on absolute mobility rates and on absolute downward occupational mobility in particular.

Lately, several researchers indeed observed that recent cohorts of individuals are more likely to be downward mobile. In the United Kingdom, the number of upward mobile persons is decreasing, whereas the number of downward mobile persons is increasing over time (Goldthorpe and Jackson 2007; Li and Devine 2011). These trends were also noticed for France (Peugny 2007). For the Netherlands, Tolsma and Wolbers (2010) found that the likelihood of downward mobility has increased for the youngest (male) birth cohorts. Particularly, men are more likely to experience downward educational and occupational mobility, whereas similar findings were not found for women.

These findings make clear that downward social mobility is (will be) more present in (future) Western societies than before. Previous social stratification research, however, has paid little attention to test (individual) explanations of downward mobility (for exceptions, see Alm 2011; Peugny 2007). This article tries to fill in this knowledge gap by testing three theoretical perspectives that aim to explain intergenerational downward occupational mobility. So, our focus is rather modest: we do not intend to give reasons why downward mobility is increasing over time (this could be the next step for future research), but first to investigate the determinants of downward mobility. In addition, we study whether the importance of these determinants has changed over time. Therefore, our research questions are as follows: *Which determinants underlie intergenerational downward occupational mobility of individuals in the Netherlands?* And: *To what extent have effects of the underlying determinants of intergenerational downward occupational mobility in the Netherlands changed over time?*

The study of intergenerational downward mobility is relevant as downward mobility may have negative consequences for an individual’s well-being. First of all, in the past scholars argued that both upward and downward mobility lead to feelings of anomy (Breed 1963; Lipset and Bendix 1959).^1^ In addition, downward mobility is an important cause of status anxiety (Layte 2012; Wilkinson 1999). Furthermore, earlier research demonstrated that downward mobility negatively affects social trust, subjective well-being and mental health (Tolsma and Wolbers 2010; Dolan and Lordan 2013; Tooth and Mishra 2013). So, in analysing the determinants of downward mobility, we obtain some evidence on how (lacking) social mobility can play an intermediating role between relevant background characteristics of individuals and their well-being.

## Theoretical Background

### Determinants of Downward Mobility

Although social stratification research has paid little attention to explanations of downward mobility, three theoretical perspectives have been developed, namely: the social casualty perspective, the meritocratic perspective and the parental resources approach. The social casualty perspective (Richardson 1977) assumes that downward mobility is mainly the result of fate. Individuals who face physical or mental problems are to a lesser extent able to attain a similar social position as their parents than healthy persons. This also applies for individuals who are addicted to drugs or alcohol, or have experienced familial disorganization in their youth. Several findings provide support for this perspective. Firstly, children of divorced parents were found to perform worse in education and are more likely to be unemployed in later life (McLanahan and Sandefur 1994). Presumably, they are therefore also more likely to be downward mobile. Secondly, Alm (2008) observed a significant association between registered drug use and downward mobility for men in Sweden. Thirdly, it appears that persons with a mental disease like schizophrenia perform worse in the labour market, and, as a consequence, are more likely to experience downward mobility (Timms 1998). Hence, we hypothesize: *(H1) Individuals with (psychical or mental) health problems are more likely to be intergenerational downward mobile.* And: *(H2) Individuals who experienced familial disorganization in early life are more likely to be intergenerational downward mobile.*

The second perspective, the meritocratic perspective (Blau and Duncan 1967; Miller 1960; Saunders 1997), is based on the idea that societies have become more open in recent decades. According to this perspective, occupational status attainment is not associated (anymore) with ascribed characteristics, like social origin, but instead, it is based on an individual’s ability and motivation (Blau and Duncan 1967). As a result, people with more ability and motivation will attain higher occupational positions. To explain this process, human capital theory provides relevant insights (Becker 1964). Human capital represents someone’s knowledge and skills and therefore his or her labour market productivity. According to this micro-economic theory, individuals who possess more human capital are considered to be more attractive employees to be hired and will therefore find themselves in better labour market positions. Besides cognitive skills, educational attainment is an important recruitment indicator for employers to signal someone’s ability and motivation (Arrow 1973). When individuals do not have the cognitive skills or educational credentials required to maintain their parents’ occupational position, they have higher risks of being downward mobile. For Great Britain, for example, several scholars discovered that people’s ability and motivation are important predictors of their opportunities for social mobility (Deary et al. 2005; Saunders 1997). Moreover, a meta-analysis of Strenze (2007) revealed that intelligence is a powerful predictor of later educational and occupational success. Dutch studies, in addition, showed that educational attainment is the most important determinant of a person’s occupational position (de Graaf and Luijkx 1995; Ganzeboom and Luijkx 2004). As a result, we expect that an individual’s cognitive skills and educational attainment provide a reduction of the risk of downward mobility. Therefore, we expect: *(H3) Individuals with more cognitive skills are less likely to be intergenerational downward mobile.* And: *(H4) Individuals with higher educational attainment are less likely to be intergenerational downward mobile.*

The third perspective, the parental resources approach, is based on the cultural reproduction theory of Bourdieu (1973). This theory argues that parents attempt to transmit their position to their children to avoid downward mobility by using various forms of (parental) resources. First of all, parents in high social classes possess more economic resources. These economic resources give children opportunities to attend better schools and provide them better educational means. Consequently, children will have better opportunities to obtain higher qualifications and, hence, better occupational positions (Blanden and Gregg 2004; de Graaf et al. 2000). With respect to downward mobility, we therefore also expect: *(H5) Individuals with more parental economic resources are less likely to be intergenerational downward mobile.*

Furthermore, children raised up in high social classes possess more cultural resources. Cultural resources enhance people’s chances of educational and occupational success and may in turn reduce the risk of downward mobility (Bourdieu 1973; Scherger and Savage 2010). There are several reasons for this claim. First of all, children are brought up in an environment that develops a greater taste to learn abstract and intellectual concepts. In addition, parents show more interest in children’s schooling performances and children receive more encouragement to attain higher educational credentials (de Graaf et al. 2000; de Graaf and Kalmijn 2001). Moreover, some scholars suggest that children with more parental cultural capital are favoured by teachers and employers, because they are familiar with the habits and customs of the elites (Dronkers and de Graaf 1995; Rivera 2012). Consequently, hypothesis 6 reads: *(H6) Individuals with more parental cultural resources are less likely to be intergenerational downward mobile.*

Lastly, the possession of parental social resources might reduce the risk of downward mobility. In their search for a job, children can use the contacts and the social network of their parents (Lin et al. 1981). As a result, children with more parental social resources might find better jobs than children with less parental social resources. In the Netherlands, research demonstrated that parental voluntary association membership is a significant predictor of intergenerational transmission of social status (van Houten et al. 2013). Moreover, close contacts between parents and teacher may stimulate the educational career of children and, in turn, their future career prospects (Dronkers and de Graaf 1995; McLean 1999). Hence, we hypothesize: *(H7) Individuals with more parental social resources are less likely to be intergenerational downward mobile.*

### Changes in Effects over Time

Several studies found that Dutch society has become more open in the sense that a process from ascription to achievement has taken place (de Graaf and Luijkx 1995; Ganzeboom and Luijkx 2004). Over time, ascribed characteristics have lost importance, whilst achieved characteristics, like education, have become increasingly important in the intergenerational transmission of occupational status (Blau and Duncan 1967). On the one hand, the upgrading of the occupational structure has led to replacement of lower skilled work by more technological advanced occupations for which employees require more skills, and education and training. As a result, the demand for high skilled workers has increased at the expense of the demand for the less educated. On the other hand, over time social norms have changed in such a way that it has become unacceptable to assess people on their social background (Parson 1951). Hence, we expect that parental resources have lost importance in explaining downward mobility, whereas achieved characteristics have become more important. Therefore our final hypotheses read: *(H8) Individuals raised in younger birth cohorts profited less from parental resources in preventing intergenerational downward mobility than individuals raised in older birth cohorts.* And: *(H9) Individuals raised in younger birth cohorts profited more from cognitive skills and educational attainment in preventing intergenerational downward mobility than individuals raised in older birth cohorts.*

## Data and Measurement

### Data

To test the predictive validity of these theoretical perspectives we used data from the Family Survey Dutch Population 2009 (Kraaykamp et al. 2009). The survey contains retrospective information on the life course and life situation of persons in the Netherlands. The target population was the total Dutch speaking population aged 18–70 in 2009. Interviews were conducted with 3269 persons. The survey consisted of two different parts: an oral questionnaire and a web/written questionnaire. In total, 2802 respondents completed both parts. The final response rate was 42.3 %.^2^

We performed several restrictions for our data analysis. First, to make a genuine cohort comparison—in order to ascertain trends in downward mobility—we account for confounding age or life course effects. In doing so, we selected only respondents who were 35 years or older and who had a job at the moment of the interview. Indeed, prior research showed that most people reach occupational maturity around the age of 35 (Goldthorpe 1980; Wolbers et al. 2011). As a result of this selection, we lose a large number of respondents, particularly women. Finally, we only include respondents with valid scores on all variables. Our final, analytical sample consists of 1423 respondents.

### Dependent Variable

The measurement of intergenerational downward mobility is based on the status of the respondent’s occupation at age 35 and the occupational status of the father when the respondent was 15 years old. The status scale is based on the International Socio-Economic Index of Occupational Status (ISEI) (Ganzeboom et al. 1992). Downward mobility is constructed by dividing the occupational status of the respondent by that of the father. The obtained scores are classified into three groups: intergenerational downward mobile persons (score < 0.9), intergenerational stable persons (0.9 <= score <= 1.1) and intergenerational upward mobile persons (score > 1.1).

Table 1 presents the percentage of persons who experienced downward mobility, stability, or upward mobility for successive birth cohorts. It appears that the percentage of downward mobile persons increased. In cohort 1925–1944 28.8 % is downward mobile, and in the youngest cohort (1965–1974) 32.5 %. The percentage of intergenerational stable or upward mobile persons, however, decreased. In the oldest birth cohort 1925–1944 24.4 % is intergenerational stable and 46.8 % is upward mobile, whereas in cohort 1965–1974 these percentages appear to be slightly lower.

**Table 1:** Developments in intergenerational occupational mobility by birth cohort and gender

Cohort	N	Downward mobile (%)	Stable (%)	Upward mobile (%)
*Total*				
1925–1944	156	28.8	24.4	46.8
1945–1954	557	30.5	24.8	44.7
1955–1964	548	32.1	22.6	45.3
1965–1974	510	32.5	23.3	44.1
Total	1771	31.5	23.7	44.1
*Men*				
1925–1944	126	25.4	25.4	49.2
1945–1954	395	26.8	27.6	45.6
1955–1964	325	28.3	22.2	49.5
1965–1974	277	31.0	26.4	42.6
Total	1123	28.1	25.5	46.4
*Women*				
1925–1944	30	43.3	20.0	36.7
1945–1954	162	39.5	17.9	42.6
1955–1964	223	37.7	23.3	39.0
1965–1974	233	34.3	19.7	45.9
Total	648	37.2	20.5	42.3

*Source*: Family Survey Dutch Population 2009 (own calculations)

In addition to general trends, we also analysed these trends for men and women separately (see Table 1). In line with earlier findings (Tolsma and Wolbers 2010), we observe that especially men are more likely to be downward mobile over time. In the oldest cohort for men 25.4 % experienced downward mobility, whereas this percentage is 31.0 % in the youngest cohort. Likewise, the percentage of intergenerational stable persons slightly increased. By contrast, the proportion of upward mobile men decreased. In the oldest cohort 49.2 % experienced upward mobility, in the youngest cohort this is only 42.6 %. Our results among women differ considerably from those among men. Table 1 reveals that the proportion of women experiencing downward mobility has decreased over time. In the oldest cohort 43.3 % is downward mobile, in the youngest cohort this percentage is only 34.3 %. In addition, the percentage of women that experienced no social mobility lies around 20 %. Finally, we notice that women in more recent birth cohorts are more likely to experience upward mobility. This percentage increased from 36.7 % in cohort 1925–1944 to 45.9 % in cohort 1965–1974. Presumably, these deviating findings for women may result from their sharply increased educational attainment and, in turn, increasing levels of labour market participation.

### Independent Variables

We study the social casualty perspective by using four characteristics mainly preceding the moment of labour market entry. Firstly, we use a measure for health, since health problems may hinder later labour market success. On a scale that varied between 1 (‘very bad’) and 5 (‘very good’), people indicated how healthy they were at the age of 20. Several studies showed that subjective measurements of health are good predictors for overall (both mental and psychical) health status (Idler and Benyamini 1997; Jylhä 2009). Secondly, various forms of family disorganization may influence the likelihood of downward mobility. We consider the effects of parental divorce and premature death of at least one of the parents. In doing so, we constructed two dummy variables. One captures whether or not a respondent experienced a parental divorce before the age of 18 (0 = no parental divorce, 1 = parental divorce), the other whether or not a respondent lost at least one of his or her parents before the age of 18 (0 = no premature death of parent, 1 = premature death of parent). Lastly, we investigate the extent to which victimization of violence is associated with a higher likelihood of experiencing downward mobility. We distinguish between whether or not a person was victim of violence before the 18th birthday (0 = no victim, 1 = victim).

The meritocratic perspective is operationalized by measurements for cognitive skills and educational attainment. Cognitive skills are measured using two questions. On a scale that varied between 1 (‘very weak’) and 5 (‘very strong’), people had to indicate whether they were good in (1) arithmetic and in (2) language at primary school. We combined these two measures to create a Likert Scale for cognitive skills (Spearmen-Brown = 0.535). Higher scores indicate more cognitive skills. Level of education is measured as the number of years required to successfully complete an education level. Scores on this variable vary between 6 (primary education) and 21 (post-academic education).

We have no direct information on the financial resources of the parents. Therefore, we base our measurement of parental economic resources on items about the presence of five luxury goods (car, microwave oven, bathroom, freezer and dishwasher) in the parental home when the respondent was 15 years old.^3^ We created a Likert Scale whereby higher scores indicate more economic resources (α = 0.662). Cultural resources are measured by questions about parental highbrow cultural participation (e.g. visiting museums and theatres), parental reading preferences (e.g. reading novels) and parental reading socialization practices when the respondent was 15 years old. Based on these items, we created a Likert Scale (α = 0.871) whereby higher scores indicate more cultural resources. Finally, social resources are measured by questions about parents’ participation rate in formal voluntary organizations like political parties, schools, religious organizations and (other) leisure organizations (among others, Rotary club, Lion’s, sport clubs). Based on these four items a sum scale (α = 0.298) is constructed. Higher scores indicate more social resources.

### Control Variables

In the analysis we take several control variables into account. First of all, we include the occupational status of the father to control for floor and ceiling effects. In addition, we control for the age of the father when the child was 15 year old to correct for life course effects of the father. Finally, we add birth cohort (in years, centred around 1957), ethnicity (0 = native, 1 = non-native), and gender (0 = female, 1 = man) as covariates in the multivariate analysis.

Descriptive statistics of the independent variables are displayed in Table 2.

**Table 2:** Descriptive statistics of independent variables (N = 1423)

Variable	Minimum	Maximum	Mean	SD
Birth year	1928.00	1974.00	1957.45	9.49
Occupational status father	24.00	86.00	45.10	14.33
Age father (at age 15 respondent)	30.00	77.00	47.18	6.31
Non-native	0.00	1.00	0.09	
Male	0.00	1.00	0.63	
Health at age 20	3.00	5.00	4.55	0.57
Parental divorce before the age of 18	0.00	1.00	0.04	
Death of a parent before the age of 18	0.00	1.00	0.06	
Victim of violence before the age of 18	0.00	1.00	0.06	
Cognitive skills	1.00	5.00	3.55	0.84
Level of education	6.00	21.00	13.08	3.39
Parental economic resources	0.00	1.00	0.34	0.27
Parental cultural resources	1.00	2.79	1.57	0.36
Parental social resources	0.00	4.00	1.02	0.99

*Source*: Family Survey Dutch Population 2009 (own calculations)

## Results

To test the determinants of intergenerational downward social mobility, we used multinomial logistic regression analysis, as our dependent variable consists of three categories (downward mobile, stable, and upward mobile persons). In the models (see Table 3 for the results) we estimated the probability of being downward mobile versus the probability of being stable. The probability of being upward mobile (versus being stable) was simultaneously estimated, but is not shown in Table 3. For interpretation purposes, we present for each independent variable the marginal effect at the mean (MEM). This marginal effect reflects the change in the probability of being downward mobile when the independent variable increases by one unit, with the other independent variables fixed on their mean values. In total, five models were estimated in which we all take into account the set of control variables. In Model 1 we include the variables of the social casualty perspective. In Model 2 we study the effects of cognitive skills and educational attainment. The effects of parental economic, cultural, and social resources are investigated in Model 3. In Model 4 we simultaneously estimate the effects of all independent variables. Finally, in Model 5 we add the interaction effects between the independent variables and birth year. To ascertain that multicollinearity does not bias our results, we first included these interaction terms separately. The effects found do not differ from the results presented here.

**Table 3:** Results of multinomial logistic regression analysis of intergenerational downward occupational mobility: downward mobile versus stable (N = 1423)

Variable	Model 1		Model 2		Model 3		Model 4		Model 5	
	MEM	SE	MEM	SE	MEM	SE	MEM	SE	MEM	SE
Birth year (1957 = 0)	−0.114	0.070	−0.014	0.071	−0.008	0.081	0.048	0.083	−0.197	0.288
Occupational status father	1.148**	0.069	1.442**	0.078	1.129**	0.077	1.506**	0.084	1.514**	0.084
Age father (at age 15 respondent)	0.100	0.108	0.090	0.105	0.103	0.105	0.082	0.108	0.072	0.108
Non-native	−0.010	0.048	−0.009	0.049	−0.001	0.048	0.000	0.050	−0.002	0.050
Male	−0.112**	0.029	−0.107**	0.029	−0.115**	0.029	−0.107**	0.029	−0.112**	0.029
Health at age 20	−0.048	0.048					−0.043	0.049	−0.074	0.164
Parental divorce before the age of 18	0.021	0.068					−0.012	0.071	−0.073	0.325
Death of a parent before the age of 18	0.074	0.058					0.018	0.060	−0.203*	0.196
Victim of violence before the age of 18	0.071	0.056					0.060	0.059	−0.055*	0.247
Cognitive skills			−0.278**	0.069			−0.262**	0.070	−0.591	0.231
Level of education			−0.638**	0.070			−0.598**	0.072	−0.531	0.216
Parental economic resources					−0.087	0.060	−0.057	0.061	−0.126	0.188
Parental cultural resources					−0.314**	0.080	−0.130	0.083	0.050	0.274
Parental social resources					−0.039	0.061	−0.012	0.062	0.045	0.197
Health at age 20 * birth year									0.052	0.244
Parental divorce before the age of 18 * birth year									0.086	0.412
Death of a parent before the age of 18 * birth year									0.382	0.318
Victim of violence before the age of 18 * birth year									0.162	0.333
Cognitive skills * birth year									0.525	0.344
Level of education level * birth year									−0.127	0.343
Parental economic resources * birth year									0.107	0.263
Parental cultural resources * birth year									−0.274	0.411
Parental social resources * birth year									−0.089	0.302
Change in Chi^2^	541.422**		693.495**		565.006**		714.356**		724.960**	
Degrees of freedom	18		14		16		28		46	

*Source*: Family Survey Dutch Population 2009 (own calculations)

* *p* < 0.05; ** *p* < 0.01 (2-sided)

The results in Model 1 indicate no significant effects of the variables of the social casualty perspective. As a result, H1 and H2 do not find empirical support. In Model 2 we examine the influence of the meritocratic perspective. This model, first of all, reveals that the probability of being downward mobile is smaller in case individuals have more cognitive skills. Likewise, it appears that as people are higher educated, they are less likely to experience downward mobility. Hence, these results support H3 and H4. The parental resource approach assumes that individuals with more parental economic, cultural and social resources are less likely to be downward mobile. However, we observe that the likelihood of being downward mobile is only lower for individuals with more cultural resources (see Model 3). These findings are, therefore, in line with H6. In Model 4 we simultaneously analyse the effects of the three theoretical perspectives. Particularly, these results provide strong support for the meritocratic perspective: higher educational credentials and more cognitive skills reduce the risk of downward mobility. Similarly, we notice that the effect of parental cultural resources disappears (that is, becomes indirect) when including the predictors of the meritocratic perspective. Finally, in Model 5 we do not find significant interaction effects between birth year and the predictors of the three theoretical perspectives on the probability to be downward mobile. Hence, these results do not support H8 and H9 that predicted changes over time in the effect of the characteristics of the meritocratic perspective and the parental resources approach.

To check the robustness of our results, we specified several alternative models. Firstly, we examined whether we can replicate our results when we do not account for floor and ceiling effects. After excluding occupational status of the father from the model, all significant effects disappear (or become positive for the variables of the parental resources approach). These results imply that in order to get adequate estimates, it is important to account for occupational status of the father. Secondly, we run separate models for individuals whose father had a high, middle, or low status job (classification of the first and the latter group are based on one standard deviation above or below the mean) to study whether the results are different for individuals of various social origins. These alternative findings do not substantially differ from the ones presented in Table 3 and, likewise, indicate that educational attainment is the most important predictor of downward social mobility. Thirdly, we tried to replicate our results by using alternative measures for occupational status: the EGP class scheme (Erikson et al. 1979) and the Dutch Ultee and Sixma occupational prestige scale (Sixma and Ultee 1983). Once again we find that our main results are robust: educational attainment is the most important determinant of intergenerational downward mobility. However, the effect of cognitive skills is weaker. Finally, we performed our analyses for men and women separately. For men the results are highly similar to the findings presented in Table 3. Most important predictor is educational attainment, followed by cognitive skills. Furthermore, these results demonstrate that a better health is negatively associated with downward mobility. This effect disappears though, when including other variables in the model. For women, we find that the loss of a parent is positively associated with the risk of intergenerational downward mobility. Yet, this effect also disappears after inclusion of other predictor variables. Notably, also in these analyses for women our results reveal significant negative effects for educational attainment and cognitive skills on the likelihood of being downward mobile. Lastly, it appears that parental economic resources protect women against downward mobility.

## Conclusion and Discussion

In the 1990s already, a few Dutch sociologists predicted that rates of intergenerational downward mobility would considerably increase in the Netherlands, but also in other Western societies (Dronkers 1994; Waslander and Bosman 1997). Observations that changes in the occupational structure did not keep pace with the educational expansion provided support for this view. As a result of ‘credential inflation’ and ‘crowding-out’ in the labour market, more and more individuals are forced to take lower social positions than their parents. Recently, various studies indeed found that individuals from younger birth cohorts are more likely to experience downward mobility than those from older cohorts (Goldthorpe and Jackson 2007; Li and Devine 2011; Peugny 2007; Tolsma and Wolbers 2010). An important follow-up question then is to investigate whether and why some individuals are more likely of being downward mobile than others. This article attempted to shed more light on this issue by studying (changes in) the determinants of intergenerational downward occupational mobility in the Netherlands.

We derived insights from three theoretical perspectives to explain why some individuals are more likely to be downward mobile: the social casualty perspective, the meritocratic perspective, and the parental resources approach. The first perspective argues that downward mobility is the result of ‘fate’. Health problems or familial disorganization in youth are expected to lead to higher probability of experiencing downward mobility. The meritocratic perspective and the parental resources approach posit rivalling hypotheses: whereas the former assumes that downward mobility is the result of selection on achievement, the latter maintains that social origin has still an important impact on someone’s future career prospects and, in turn, on someone’s risk of downward social mobility.

The empirical results provide little support for the social casualty perspective. Possibly, the lack of empirical support for this perspective stems from the fact that these events are quite rare or that more extreme cases felt outside our sample population or tend to have higher non-response rates. Accordingly, our results could be an underestimation of the true importance of the social casualty perspective.

Similarly, the parental resources approach is not clearly supported by our findings. Although we do find that parental cultural resources provide some protection against downward mobility, this effect largely becomes indirect when we take into account other characteristics of individuals.

The meritocratic perspective, by contrast, is more important in explaining downward mobility for individuals. As individuals are higher educated or have more cognitive skills, they are less likely to be downward mobile. These results are much in line with previous results found in France (Peugny 2007) and Sweden (Alm 2011). However, in contrast to our expectations, we did not find that the meritocratic perspective has gained more importance over time. Thus, despite the fact that Dutch society has become more open and achieved characteristics have become more important than ascribed characteristics in attaining high social positions (de Graaf and Luijkx 1995), education and cognitive skills have not become more important in preventing intergenerational downward occupational mobility.

Consequently, one of our main conclusions is that downward mobility is mainly the result of the meritocratic principle selection on effort and ability. Accordingly, our results may imply that despite the expected increase in the number of downward mobile individuals, this trend will not affect the efficiency of the labour system. Yet, because studies have shown that children from higher social strata enter secondary education with a knowledge advantage (Kloosterman et al. 2011) they might avert the risk of downward mobility in earlier life phases so that inequalities may still be persistent over time. In addition, the results clearly demonstrate that, despite processes of ‘credential inflation’ and ‘crowding-out’ in the labour market, individual investment in education remains profitable: indeed, these investments reduce the personal risk for intergenerational downward mobility. Thus, from a general point of view, the findings indicate that educational credentials are not so much of importance in absolute, but especially in relative terms. Another interpretation of the results might be that the emergence of a homogenous underclass of lower educated has become ever more likely (Gesthuizen and Kraaykamp 2002). Because talented persons from lower social backgrounds have profited from the educational expansion, those left behind, including downward mobile persons, are the ones with least cognitive skills (and qualifications). And, as a result of this development, especially this vulnerable group, but also upcoming generations of this group, may hold more marginalised positions with all negative consequences (from anomy to mental health problems) associated (Wilson 1987).

Although this article provides new insights into the individual determinants of downward mobility, some aspects deserve more scholarly attention in future research. First of all, in this article we focused on individual explanations of intergenerational downward occupational mobility. However, the effects of structural changes, such as cyclical fluctuations of the economy, could be further investigated. In light of the gender differences found, scholars may also investigate to what extent the risen labour market participation of women has had an effect on the risk of downward mobility of men, due to increased direct competition among scarce jobs between men and women. In addition, although studies in several countries showed that rates of downward mobility are rising, comparative research is still lacking. To fully understand the causes of downward mobility, cross-national research is therefore required. Finally, in this article we investigated intergenerational downward occupational mobility as a result of a two-generation process. However, as proposed by the status consolidation theory of Richardson (1977), a three-generation approach might be more appropriate. Specifically, this theory argues that parents, who experienced upward social mobility in the past without a comparable increase in their educational attainment, cannot provide their children enough parental resources to maintain their social position. As a result, their children will end up in the same social positions as their grandparents did, thereby consolidating social status across three generations. To stringently test this hypothesis, social stratification and mobility research with a focus on three generations is necessary (Mare 2011). In the Netherlands, a first step into this direction has been made recently (Wolbers and Ultee 2013).

## Footnotes

1 Recent empirical evidence does not strongly support this claim anymore with regard to upward mobility (Clark and D’Angelo 2013).

2 Given that interviews were time-consuming (approximately 45–60 min) and both the respondent and his or her partner were interviewed, the response rate can be considered as relatively high (and very similar to response rates for earlier rounds of the Family Survey Dutch Population). Moreover, the response rate did not lead to any known response bias (Kraaykamp et al. 2009).

3 We acknowledge that this measure of parental economic resources does not fully capture the financial capacities of parents, especially for people in younger birth cohorts. Yet it is important to keep in mind that we investigate respondents who are at least 35 years old. Accordingly, the situation asked refers to the possession of multiple “luxury goods” of parents before 1990. Nevertheless, the validity of this measure over time may still be doubtful, but a better measure of parental economic resources is simply not available in the data. Anyway, excluding parental economic resources from our models, did not lead to different results.
